# Early Immune Initiation by Porcine Cells following *Toxoplasma gondii* Infection versus TLR Ligation

**DOI:** 10.3390/microorganisms9091828

**Published:** 2021-08-28

**Authors:** Benjamin Hamid, Josephine Schlosser-Brandenburg, Lalita Bechtold, Friederike Ebner, Sebastian Rausch, Susanne Hartmann

**Affiliations:** Institute of Immunology, Department of Veterinary Medicine, Freie Universität Berlin, 14163 Berlin, Germany; benjamin.hamid@fu-berlin.de (B.H.); josephine.schlosser@fu-berlin.de (J.S.-B.); l.bechtold@fu-berlin.de (L.B.); friederike.ebner@fu-berlin.de (F.E.); sebastian.rausch@fu-berlin.de (S.R.)

**Keywords:** dendritic cells, IFNγ, IL-12, IL-18, monocytes, NK cells, NKp46, pigs, TLR, *Toxoplasma gondii*

## Abstract

Containment of acute *Toxoplasma gondii* infection is dependent on an efficient interferon gamma response. However, the earliest steps of immune response initiation immediately following exposure to the parasite have not been previously characterized in pigs. Murine and human myeloid cells produce large quantities of interleukin (IL)-12 during early *T. gondii* infection. We therefore examined IL-12 expression by porcine peripheral blood monocytes and dendritic cell (DC) subsets following toll-like receptor (TLR) ligation and controlled *T. gondii* tachyzoite infection. We detected IL-12p40 expression by porcine plasmacytoid DC, but not conventional or monocyte-derived DC following TLR ligation. Unexpectedly, we also observed considerable IL-12p40 production by porcine CD3– NKp46+ cells—a classical natural killer cell phenotype—following TLR ligation. However, in response to *T. gondii* exposure, no IL-12 production was observed by either DC or CD3– NKp46+ cells. Despite this, IL-18 production by DC-enriched peripheral blood mononuclear cells was detected following live *T. gondii* tachyzoite exposure. Only combined stimulation of porcine peripheral blood mononuclear cells with recombinant IL-12p70 and IL-18 induced innate interferon gamma production by natural killer cells, while T cells and myeloid cells did not respond. Therefore, porcine CD3– NKp46+ cells serve as important IL-12 producers following TLR ligation, while IL-18 likely plays a prominent role in early immune response initiation in the pig following *T. gondii* infection.

## 1. Introduction

*Toxoplasma gondii* is an extremely widespread protozoan parasite, with a recent large meta-analysis examining data collected from pregnant women suggesting that 31.8–35.9% of this subgroup are seropositive for *T. gondii* globally [1]. The major routes of human infection include consumption of water or raw vegetables which have been contaminated with oocysts originating from cat feces, or consumption of raw or undercooked meat containing tissue cysts [2]. Pork and mutton are the meats most commonly associated with transmission of *T. gondii* via tissue cysts, with another meta-analysis finding 12.3% of pork and 14.7% of mutton samples positive for *T. gondii* [2,3]. Similarly, 41% of foodborne toxoplasmosis cases in the United States have been found to be associated with consumption of infected pork [4]. Despite the importance of *T. gondii* infections in pigs for public health, the porcine immune response to the parasite is relatively understudied.

In mice and humans, interferon gamma (IFNγ) is the primary driver of resistance to acute *T. gondii* infection [5,6]. Similarly, Rahman et al. (2020) [7] demonstrated that peripheral blood mononuclear cells (PBMCs) isolated from *T. gondii*-infected pigs produce IFNγ upon antigen-specific restimulation, beginning 8 days post-infection, and peaking 14 days post-infection. This confirms the presence of an antigen-specific T-cell response in pigs to *T. gondii*. However, the earliest steps of immune initiation, which occur immediately following exposure and lead to the polarization of T helper cells towards a type 1 (Th1) phenotype, remain unclear.

In mice and humans, dendritic cells (DC) and monocytes begin to produce interleukin (IL)-12 shortly after infection [5,6]. In mice, this is mediated via the MyD88 pathway [8] through recognition of the soluble *T. gondii* antigen profilin by toll-like receptors (TLR) 11 [9] and 12 [10]. In humans, the recognition pathway is unclear, but initiation requires phagocytosis of a live tachyzoite [6,11]. IL-12 production does not occur when human cells are stimulation with heat-killed *T. gondii* tachyzoites, or when phagocytosis or endosomal acidification are inhibited [11]. Thus, the mechanisms of parasite recognition differ greatly between mice and humans; however, in both cases, IL-12 is ultimately produced in large quantities by myeloid cells [6]. This myeloid-cell-derived IL-12 stimulates natural killer (NK) cells and CD8+ T cells to release IFNγ, as well as the differentiation of CD4+ T cells into Th1 cells, which further secrete IFNγ, among other factors [5,6,12]. An influx of IL-12+ neutrophils has also been described in the mouse following intraperitoneal *T. gondii* infection [13]; however, in contrast to DC [14] and monocytes [15], neutrophils are not essential for controlling oral *T. gondii* infection [15].

Once the immune response has been mounted, in order to escape destruction, *T. gondii* tachyzoites convert into much more slowly replicating forms called bradyzoites. These bradyzoites can persist in cyst-like structures within the brain, muscles, and other organs for life, and can transform back into tachyzoites should the host lose immunocompetence [5,16].

In recent years, significant differences have been identified between the early immune responses of mice and humans to *T. gondii*. Notably, the subsets of monocytes and DC that produce IL-12 following infection are reversed. In mice, it is inflammatory monocytes and type 1 conventional DC (cDC1) that are responsible for IL-12 secretion, while the human analogues of these cells do not respond. Instead, human nonclassical and intermediate monocytes and type 2 conventional DC (cDC2) respond with IL-12 production [6,11].

The pig is often considered to be immunologically human-like, with genomic studies indicating that 80% of porcine immune-response genes resemble human equivalents, compared to less than 10% in mice [17,18]. In pigs, as in humans, postnatal *T. gondii* infections usual remain asymptomatic, but congenital toxoplasmosis can cause severe pathology. This is in contrast to mice, in which infection with some *T. gondii* strains can be fatal, but fetal infections are rare [19,20,21,22]. Further, like humans, pigs lack TLR 11 and 12 [23], and are therefore unable to recognize the parasite via this mechanism as mice do. These similarities hint that perhaps recognition mechanisms and early immunological responses may be similar between humans and pigs.

However, human and murine cDC have both been reported to also strongly express IL-12 in response to appropriate TLR stimulation [24]. In contrast, phenotypic analysis of porcine DC subsets by Auray et al. (2016) [25] revealed a lack of IL-12 in supernatants of cDC following in vitro stimulation with a panel of TLR ligands. The responses of each porcine DC subset to stimulation with a strongly Th1-inducing pathogen such as *T. gondii* have not been previously characterized.

Here, we aim to extend our understanding of IL-12 production by porcine myeloid cells by characterizing in parallel the responsiveness of monocytes, monocyte-derived DC (moDC), cDC1, cDC2, and plasmacytoid DC (pDC) to infection with *T. gondii* tachyzoites in comparison to TLR ligation. In addition to characterizing each subset in isolation, we also analyzed monocytes and DC during co-culture with lymphocyte subsets. This allowed us to examine the cells responsible for driving the subsequent IFNγ response during early *T. gondii* infection in pigs.

Our data underlined that porcine monocytes and cDC do not produce IL-12 following TLR ligation, leaving pDC as the only IL-12-producing myeloid PBMC in pigs. However, we identified substantial endogenous IL-12 production by circulating porcine CD3– NKp46+ cells following TLR ligation. In contrast, when exposed to live *T. gondii* tachyzoites, neither pDC nor this CD3– NKp46+ population produced detectable levels of IL-12. However, IL-18 production by DC-enriched PBMCs was detected following both TLR ligation and live *T. gondii* tachyzoite exposure. This hinted at an alternative mechanism of IFNγ induction during early *T. gondii* infection in pigs. Therefore, the initial IL-12 response of porcine PBMCs to *T. gondii* infection seems to be substantially different than that observed in humans or mice, and differs from the response to distinct TLR ligation. Porcine CD3– NKp46+ cells serve as an important IL-12 source following TLR ligation, and IL-18 likely plays a prominent role in the early immune response to *T. gondii* infection, which deserves further investigation.

## 2. Materials and Methods

### 2.1. PBMC Isolation

Whole peripheral blood was collected in 1.5 mg/mL ethylenediaminetetraacetic acid (EDTA) from 6-month-old fattening pigs upon exsanguination at the slaughterhouse. Blood was diluted 1:2 in 0.9% NaCl solution and density gradient centrifugation was performed using Pancoll (density: 1.077 g/mL; PAN-Biotech GmbH; Aidenbach, BY, Germany) to isolate the buffy coat layer. Isolated cells were washed in 0.9% NaCl, and remaining red blood cells were lysed using erythrocyte lysis solution (H_2_O with 0.01 M KHCO_3_, 0.155 M NH_4_Cl, 0.1 mM EDTA, pH 7.5) for 5 min at room temperature. Leukocytes were washed and resuspended in complete Iscove’s modified Dulbecco’s medium (cIMDM) containing IMDM, 10% fetal bovine serum (FBS), 100 U/mL penicillin, and 100 ug/mL streptomycin (all: PAN-Biotech GmbH).

### 2.2. Cell Sorting Pre-Enrichment via Magnetic Cell Separation (MACS)

Pre-enrichment was performed to facilitate cell sorting in terms of improved yield and purity. PBMCs were labelled with murine anti-pig antibodies, all immunoglobulin (Ig) G subtype 1 (IgG1), against CD3 ε, gdTCR1, NKp46, CD21, and IgM, and then with anti-mouse IgG1-microbeads (15 min, 4 °C). Bead-labelled and unlabelled cells were separated using the sensitive depletion program on the AutoMACS Pro Separator (Miltenyi Biotec; Bergisch Gladbach, NW, Germany). The negative fraction was labelled with anti-human CD14-microbeads (clone TÜK4) and MACS-sorted to obtain pure CD14+ monocytes and a CD14-depleted DC-rich fraction. For co-culture experiments, it was required to sequentially isolate CD3+ T cells and NKp46+ NK cells, which was performed by splitting up the initial MACS-sorting into multiple steps. Additional details of all antibodies used are available in Table S1.

### 2.3. Fluorescence-activated Cell Sorting (FACS)

**Figure 1:**
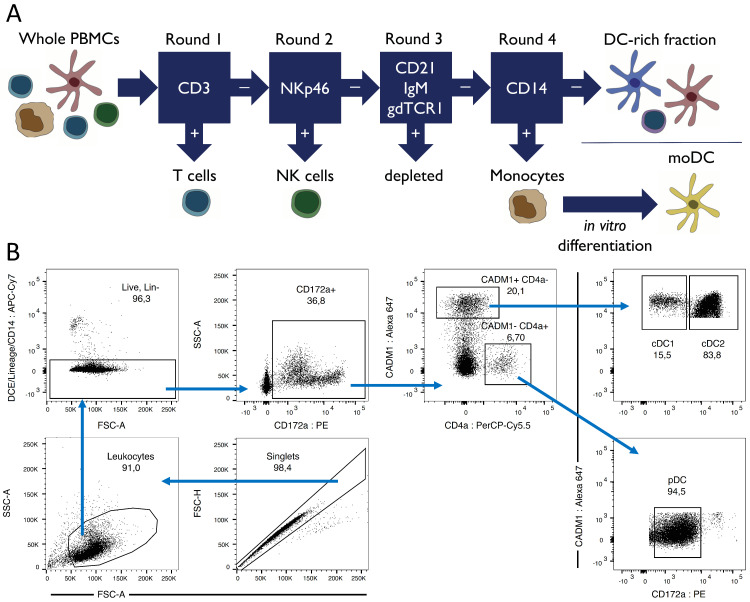
(**A**) Illustration of the magnetic-activated cell sorting (MACS) strategy used to isolate CD3+ T cells, CD3– NKp46+ cells, monocytes, and DC-enriched PBMCs. Consecutive rounds of sorting were performed to successively sort CD3+ T cells, NKp46+ cells, and CD14+ monocytes, and to further deplete CD21+, IgM+, and gdTCR1+ cells in order to obtain a DC-enriched population. Monocytes were subsequently differentiated in vitro to generate monocyte-derived (mo)DC. (**B**) FACS-sorting strategy used to isolate bona fide DC subsets from DC-enriched PBMCs. Cell aggregates were first excluded based on FSC-A/FSC-H size ratio, and then larger, less-granular cells were selected based on FSC-A/SSC-A parameters. Dead cells and any CD3+, gdTCR1+, CD21+, IgM+, NKp46+, or CD14+ cells remaining after MACS were excluded. CD172a+ cells were selected, and this population was differentiated on the basis of CD4a and CADM1 expression. CADM1+ CD4a− and CADM1− CD4a+ subpopulations were both selected and further segregated for CD172a expression. CADM1+ CD4a− CD172a^low^ cells constitute cDC1, CADM1+ CD4a− CD172a^high^ cells constitute cDC2, and CADM1− CD4a+ CD172a^mid^ cells are classified as pDC. FACS plots display forward and side-scatter values on a linear scale, while all fluorescent markers are displayed on a biexponential scale.

For further cell isolation, the DC-rich fraction was relabelled using the set of murine anti-pig antibodies described in Section 2.2, and secondary anti-mouse IgG1-APC-Cy7 (dump channel). In addition, cells were labelled with anti-pig CD14 (Clone MIL2; IgG2b) and secondary goat anti-mouse IgG2b-APC-Cy7 (dump channel), CADM1-Alexa 647, CD4a-PerCP-Cy5.5, and CD172a-PE. Fixable viability dye in eFluor^®^ 780 (ThermoFisher Scientific; Waltham, MA, USA) was used to exclude dead cells into the dump channel. Bona fide DC subsets were sorted using the FACSAria III instrument (BD Biosciences; Franklin Lakes, NJ, USA) according to the sorting strategy shown in Figure 1B. The cDC1 were defined as CADM1+ CD4a− CD172a^low^, the cDC2 were defined as CADM1+ CD4a− CD172a^high^, and the pDC were defined as CADM1− CD4a+ CD172a^mid^. Sorting was performed prior to in vitro stimulation, since CADM1 and CD4a expression were downregulated following some of the treatments, making subsequent subset identification impossible.

### 2.4. Generation of moDC from CD14+ Sorted Cells

CD14+ monocytes were seeded at a density of 1 × 10^6^ cells/mL in cIMDM supplemented with 20 ng/mL recombinant porcine granulocyte-macrophage colony-stimulating factor (GM-CSF) and 10 ng/mL recombinant porcine IL-4 (both: R&D Systems; Minneapolis, MN, USA). Cells were incubated for 7 days at 37 °C, 5% CO_2_. After 4 days, cells were washed, and media supplemented with GM-CSF and IL-4 was replaced. After 7 days, nonadherent cells were harvested, washed, and used for further applications.

### 2.5. T. Gondii Tachyzoite Culture

Hs27 human foreskin fibroblasts (ATCC CRL-1634) were cultured at 37 °C, 5% CO_2_ in fibroblast media containing high glucose Dulbecco’s modified Eagle’s medium (DMEM) with stable glutamine, 10% FBS, 100 U/mL penicillin, and 100 ug/mL streptomycin (all: PAN-Biotech GmbH). Confluent cultures were infected with green fluorescent protein (GFP)-labelled RH-strain *T. gondii* tachyzoites and incubated until most fibroblasts had lysed and extracellular tachyzoites were visible microscopically. Supernatants were removed and centrifuged at 100× *g* for 5 min to pellet large cell debris, then supernatants were transferred to new tubes and centrifuged at 300× *g* for 10 min to pellet tachyzoites. Tachyzoites were resuspended in phosphate-buffered saline (PBS), syringed through a 5 µm filter to remove additional cell debris, and viable tachyzoites were counted by trypan blue assay. Tachyzoites were pelleted and resuspended in cIMDM at the required density. Heat-killed tachyzoites were prepared after resuspension by heating to 90 °C for 15 min. Hs27 and *T. gondii* tachyzoite culture protocols were adapted from Khan and Grigg (2017) [26].

### 2.6. In Vitro TLR-ligand Stimulations and T. gondii Infections of Monocytes, moDC, cDC, pDC, T Cells, and CD3– NKp46+ Cells

Monocytes and moDC were seeded in cIMDM in 24-well plates at a density of 1 × 10^6^ cells/mL/well. Monocytes were preincubated at 37 °C, 5% CO_2_ for 24 h prior to treatment, and moDC were treated immediately following their 7 days of differentiation. The cDC1, cDC2, pDC, CD3+ T cells, and CD3– NKp46+ cells were seeded in round-bottomed 96-well plates at a density of 1 × 10^6^ cells/mL, with 200 µL/well. For co-cultures, the ration of myeloid cells to lymphocytes was standardized at 1:3, with the total cell density maintained at 1 × 10^6^ cells/mL. Cells were pelleted, supernatants were removed, and cells were resuspended in cIMDM supplemented with lipopolysaccharide (LPS; 100 ng/mL) + resiquimod (R848; 300 ng/mL), polyinosinic:polycytidylic acid (Poly(I:C); 500 ng/mL) + R848 (300 ng/mL), live *T. gondii* tachyzoites (MOI 1:1), heat-killed *T. gondii* tachyzoites (MOI 1:1), recombinant profilin (1 µg/mL), or left untreated where indicated. Cells were stimulated with LPS or poly(I:C) to simulate bacterial or viral stimulation via TLR 4 and TLR 3, respectively. Both were applied in combination with R848, a TLR 7/8 ligand that acts as an adjuvant. Following treatment, cells were incubated at 37 °C and 5% CO_2_ for 24 h.

### 2.7. FACS Analysis of GFP+ T. gondii Infection

Following tachyzoite exposure for 24 h, cells were pelleted and stained with fixable viability dye in eFluor^®^ 780 (ThermoFisher Scientific) to exclude dead cells, and then acquired using the FACS Canto instrument (BD Bioscientific).

### 2.8. Cytokine Measurements in Supernatants

Cells were incubated for 24 h, pelleted, and supernatants were collected. Supernatants were analyzed for tumor necrosis factor alpha (TNF-α; R&D Systems; lower limit of detection—31.1 pg/mL), IL-12/IL-23p40 (R&D Systems; lower limit of detection—78.1 pg/mL), or IL-18 (Merck; lower limit of detection—81.92 pg/mL) by enzyme-linked immunosorbent assay (ELISA; plate reader: BioTek Synergy H1; Winooski, VT, USA; washer: Tecan HydroSpeed; Männedorf, ZH, Switzerland).

### 2.9. FACS-analysis of Myeloid Cells after TLR-ligand Stimulation or T. gondii Infection

Following stimulation, cells were incubated at 37 °C, 5% CO_2_ for 24 h, with brefeldin A (ThermoFisher Scientific) added after 18 h to a final concentration of 3 µg/mL. Cells were pelleted and labelled with murine IgG2a isotype CD152muIg, which labels CD80 and CD86 on pig cells, followed by secondary anti-mouse IgG2a-Brilliant Violet 605. Cells were further labelled with CD172a-PE and fixable viability dye in eFluor^®^ 780 (ThermoFisher Scientific). Cells were treated with fixation/permeabilization solution according to the manufacturer’s instructions (Invitrogen; Waltham, MA, USA) and then labelled with mouse anti-pig IL-12p40-biotin and mouse anti-human TNF-α-Pacific Blue, and stained with streptavidin-PE-Cy7. Data was acquired using the FACSAria III instrument (BD Biosciences).

### 2.10. PBMC Stimulation with Recombinant IL-12 and IL-18 Stimulation, and IFNγ ELISA

PBMCs were seeded in round-bottomed 96-well plates at a density of 1 × 10^6^ cells/mL and 200 ul/well. Cells were pelleted and resuspended in cIMDM supplemented with 10, 100, or 1000 ng/mL recombinant porcine IL-12p70 (R&D Systems), each with and without 100 ng/mL recombinant porcine IL-18 (R&D Systems). Cells were also resuspended in cIMDM supplemented with 10, 100, or 1000 ng/mL recombinant porcine IL-18, each with and without 100 ng/mL recombinant porcine IL-12p70. Cells were incubated at 37 °C, 5% CO_2_ for 48 h, pelleted, and supernatants were collected. Supernatants were analyzed for IFNγ concentrations by ELISA (R&D Systems; lower limit of detection—62.5 pg/mL).

### 2.11. Determining the Cellular Source of IFNγ following IL-12/IL-18 Stimulation

PBMCs were seeded in cIMDM in T25 flasks at a density of 1 × 10^6^ cells/mL, with 5 mL/flask for 12 h at 37 °C, 5% CO_2_. Media was supplemented with 1 ug/mL recombinant IL-12p70 and 1 ug/mL recombinant IL-18, plus brefeldin A (3 ug/mL; ThermoFisher Scientific). Control flasks were prepared without cytokine treatment, with and without brefeldin A. The following antibodies were used for staining PBMC subsets: mouse anti-pig CD172a (IgG1), mouse anti-pig CD8a (IgG2a), anti-mouse IgG1-APC-Cy7, anti-mouse IgG2a-Brilliant Violet 605, mouse anti-pig CD3-PerCP-Cy5.5, mouse anti-pig CD4-PE-Cy7, mouse anti-pig CD16-FITC, mouse anti-pig CD335/NKp46-APC, and mouse anti-pig IFNγ-PE. For intracellular staining of IFNγ, cells were fixed and permeabilized with intracellular fixation buffer (eBioscience; San Diego, CA, USA). Fixable viability dye in eFluor^®^ 506 (ThermoFisher Scientific) was used to exclude dead cells. Cells were acquired using the FACSAria III instrument (BD Biosciences).

## 3. Results

### 3.1. Porcine Dendritic Cell Sorting

To isolate bona fide DC subsets for in vitro studies, freshly isolated whole PBMCs underwent multiple rounds of MACS followed by FACS-sorting, according to the sorting strategy outlined in Figure 1A,B. To verify the identity of our FACS-sorted DC, subsets were additionally analyzed for CD1 and SLA-DR expression (data not shown). The cDC1 were CD1-, while cDC2 were mostly CD1+. All cDC were SLA-DR+, while pDC exhibited heterogenous SLA-DR expression. These phenotypes corresponded with previously published data [25,27].

### 3.2. Porcine PMBC Subset Responses to TLR Ligation

First, we investigated the responsiveness of the isolated porcine cell subsets to TLR ligation to demonstrate the ability the cells to respond to stimulation.

#### 3.2.1. Monocytes and moDC Do Not Produce IL-12 following TLR Ligation

**Figure 2:**
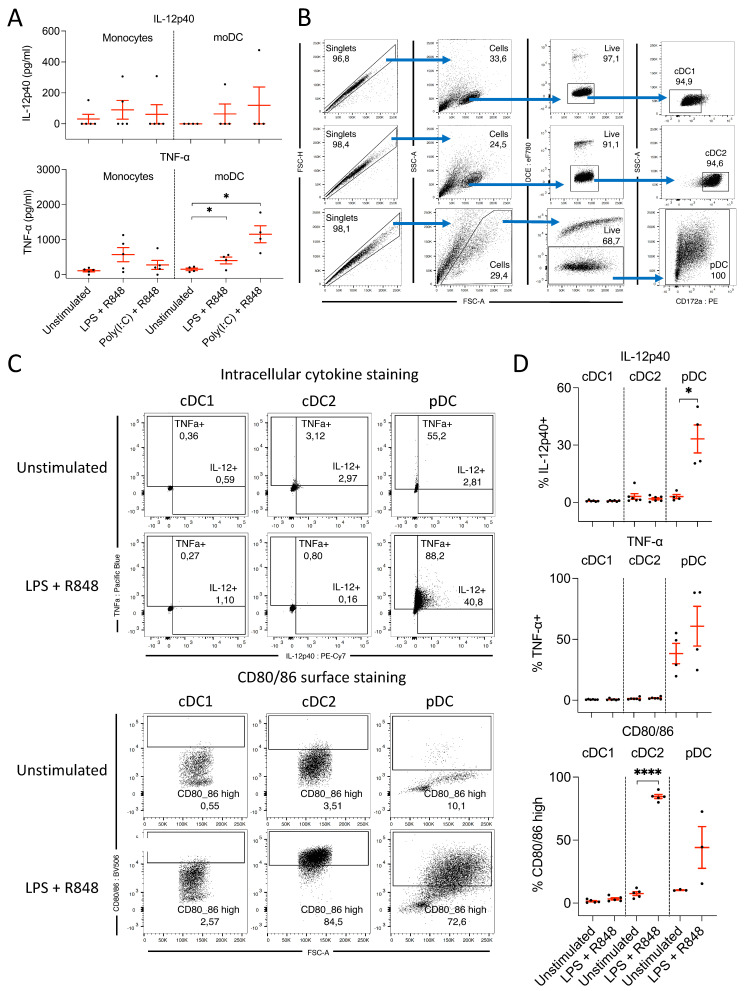
(**A**) Concentrations of IL-12p40 and TNF-α detected in monocyte and moDC cell culture supernatants by ELISA following 24 h of stimulation with different TLR ligand combinations. Both cytokines were quantified in parallel from the same supernatants. Monocyte stimulations were performed from *n* = 5 different pigs, whereas *n* = 4 moDC stimulations were performed. (**B**) Gating strategy used to select sorted bona fide DC subsets after stimulation, prior to FACS analysis of intracellular cytokine production and CD80/86 expression by staining for CD152muIg. (**C**) Representative FACS analysis of cDC1, cDC2, and pDC demonstrating induction of IL-12p40 and TNF-α production by pDC and CD80/86 expression by cDC2 and pDC following stimulation with LPS (100 ng/mL) + R848 (300 ng/mL). (**B**,**C**) FACS plots displaying forward and side-scatter values on a linear scale, while all fluorescent markers are displayed on a biexponential scale. (**D**) Summary of percentages of IL-12p40+, TNF-α+, and CD152muIg^high^ expressing cDC1, cDC2, and pDC following 24 h of stimulation with LPS (100 ng/mL) + R848 (300 ng/mL) vs. unstimulated control. Each dot represents the result from a different pig. (**A**,**D**) Asterisks indicate statistical significance; paired *t* test; * *p* ≤ 0.05, **** *p* ≤ 0.0001.

To assess the IL-12 production capacity of porcine monocytes and moDC, cells were stimulated with either LPS or poly(I:C) in combination with R848. IL-12p40 and TNF-α concentrations in cell culture supernatants were analyzed by ELISA in parallel, to compare IL-12p40 production with an alternative myeloid cell-derived inflammatory cytokine (Figure 2A).

Porcine monocytes and moDC did not substantially upregulate IL-12p40 following TLR ligation, as cell fractions from the majority of pigs did not secrete detectable levels of IL-12p40 following LPS or poly(I:C) treatments. A small upregulation of TNF-α expression by both monocytes and moDC was detected following stimulation with LPS + R848. In contrast, TNF-α concentrations in the supernatants of monocytes stimulated with poly(I:C) + R848 were similar to the negative control treatment group, but were substantially elevated in moDC. This indicated that porcine monocytes and moDC did produce low levels of TNF-α following ligation of TLR 3, 7, and 8 or 4, 7, and 8. However, the magnitude of this response and the lack of IL-12 response contrast sharply with what has been documented in other species.

#### 3.2.2. pDC, but Not cDC, Produce IL-12 following Ligation of TLR 4, 7, and 8

IL-12p40 and TNF-α production by cDC and pDC were measured by FACS following stimulation with LPS + R848 (Figure 2B). Peripheral blood cDC did not produce IL-12p40 or TNF-α following TLR ligation; however, both cytokines were induced within pDC (Figure 2C,D). Approximately 40% of pDC produced TNFa even when unstimulated, which increased to approximately 60% following treatment. Similarly, Auray et al. (2016) [25] previously found the gene encoding TNFa to be overexpressed by rested porcine pDC, and found TNFa in the supernatants of unstimulated porcine pDC. In contrast to the lack of IL-12p40 or TNFa produced by cDC, costimulatory ligand expression, represented by CD80/86, was strongly increased in the cDC2 subset. Approximately 85% of cDC2 were found to be CD80/86^high^ following stimulation, compared to <10% of untreated cDC2. No upregulation was observed within the cDC1 subset, while the pDC displayed a moderate upregulation with large interanimal variation (Figure 2C,D). Thus, pDC were found to be the only IL-12p40 and TNF-α source within the bona fide DC subsets.

An unexpected observation within the pDC subset was a large difference in cell survival by treatment group. LPS + R848-treated pDC, which upregulated IL-12p40 and TNF-α expression, survived in substantially larger numbers than those that were unstimulated (data not shown). This indicated a very short lifespan of unstimulated pDC in culture, with survival being substantially elongated following appropriate stimulation. Similarly, historical reports indicate that human cells with a phenotype corresponding to pDC underwent apoptosis rapidly following isolation, but could be rescued by administration of IL-3 [28].

#### 3.2.3. CD3– NKp46+ Cells Produce IL-12 following TLR Ligation

**Figure 3:**
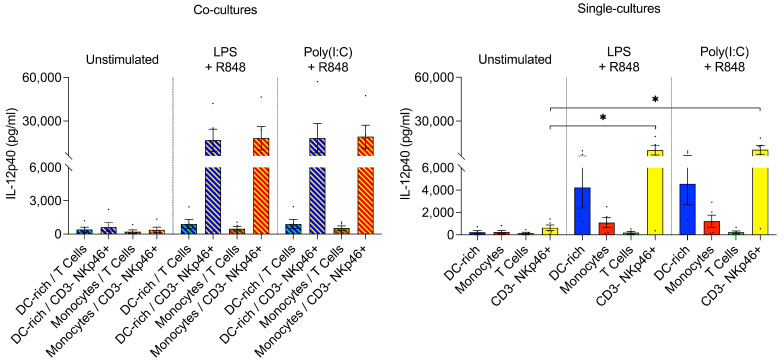
IL-12p40 concentrations in the supernatants of porcine monocytes and DC-enriched PBMCs co-cultured with T cells and CD3– NKp46+ cells (**left**), as well as in isolation (**right**). All were following 24 h stimulation with LPS (100 ng/mL) or poly(I:C) (500 ng/mL) and R848 (300 ng/mL). All cell types were stimulated in parallel from each pig, with a total of *n* = 5 pigs. Co-cultures were performed with a ratio of 1 monocyte/DC to 3 T cells/CD3– NKp46+ cells. Asterisks indicate statistical significance; paired *t* test; * *p* ≤ 0.05.

We observed a striking lack of IL-12 production by isolated porcine monocytes and moDC and cDC subpopulations following TLR ligation. We therefore speculated that additional signals are required for porcine myeloid cells to initiate IL-12p40 production. One hypothesis was that CD40 on the surface of porcine myeloid cells may require interaction with CD40L, displayed by activated T cells, to initiate an IL-12 response. To investigate this, monocytes and DC-enriched PBMCs were co-cultured with T cells that were previously activated using anti-CD3 antibodies during sorting. However, we found that IL-12p40 and TNF-α production in these co-cultures was not elevated following stimulation (Figure 3, left).

Another hypothesis was that stimulation of myeloid cells by IFNγ, provided initially by natural killer (NK) cells, was required to induce IL-12 release. Therefore, we also co-cultured monocytes and DC-enriched PBMCs with MACS-sorted CD3– NKp46+ cells (Figure 3, left). Here we found that IL-12p40 production was greatly enhanced following stimulation. However, surprisingly, we found that porcine CD3– NKp46+ cells alone produced similarly high levels of IL-12p40 following TLR stimulation (Figure 3, right). Therefore, our data indicated that CD3– NKp46+ cells serve as an alternative and previously undescribed IL-12p40 source in the pig.

### 3.3. Porcine PMBC Subset Responses to Toxoplasma gondii Infection

TLR ligation experiments demonstrated the responsiveness of our sorted monocytes and DC subsets; with monocytes and moDC upregulating TNF-α, pDC upregulating IL-12p40 and TNF-α, and cDC2 upregulating CD80/86 upon specific TLR stimulation. Next, we investigated whether stimulation with live or heat-killed *T. gondii* tachyzoites, or recombinant profilin, resulted in similar patterns of IL-12p40, TNF-α, and CD80/86 expression. In parallel, we examined whether porcine CD3– NKp46+ cells, which exhibited strong IL-12p40 production following TLR ligation, likewise responded to *T. gondii* stimulation.

#### 3.3.1. GFP-labelled *T. gondii* Tachyzoites Are Infective and Detectable by FACS

**Figure 4:**
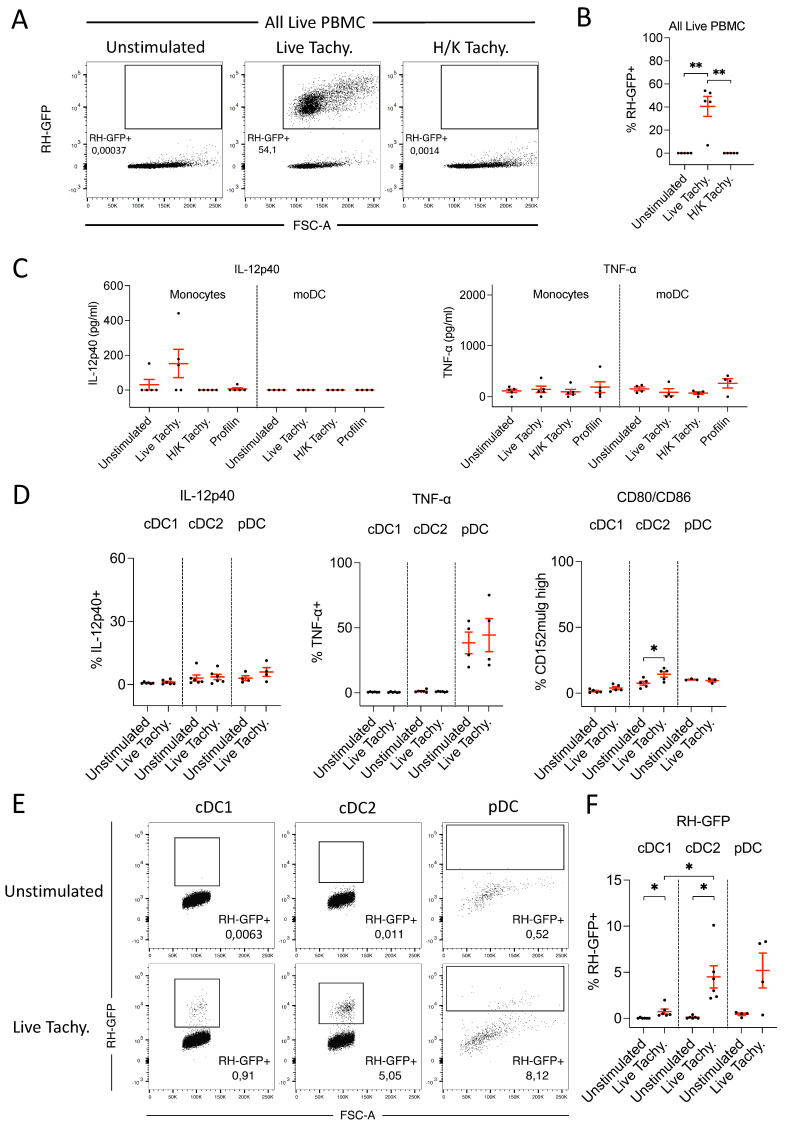
(**A**) Representative plots of GFP+ PBMCs following 24 h incubation with live or heat-killed GFP-labelled *T. gondii* tachyzoites. (**B**) Percentage of cells infected with GFP-labelled RH-strain *T. gondii* tachyzoites (RH-GFP+) within the live PBMC population following 24 h treatment with live or heat-killed tachyzoites (MOI 1:1). Data shown are PBMCs isolated from 5 different pigs. Live and heat-killed tachyzoite stimulations were performed in parallel. (**C**) Concentrations of IL-12p40 and TNF-α detected in monocyte and moDC cell culture supernatants following 24 h stimulation with live or heat-killed *T. gondii* tachyzoites, or recombinant profilin. Cytokines were quantified in parallel from the same supernatants of *n* = 4/5 pigs. (**D**) Percentage IL-12+, TNF-α+, CD80/86^high^ cDC1, cDC2, and pDC following 24 h stimulation with live *T. gondii* tachyzoites. (**E**) Representative gating demonstrating differences in the percentages of GFP+ cDC1, cDC2, and pDC following 24 h incubation with live GFP-labelled *T. gondii* tachyzoites. (**F**) Percentages of *T. gondii*-infected cDC1, cDC2, and pDC following 24 h stimulation with live *T. gondii* tachyzoites. (**A**,**E**) FACS plots displaying forward and side-scatter values on a linear scale, while all fluorescent markers are displayed on a biexponential scale. (**D**,**F**) Each dot represents the result from a different pig. Repeats were performed using cells from different pigs on different days, stimulated according to the same protocol. (**B**,**D**,**F**) Asterisks indicate statistical significance; paired *t* test; * *p* ≤ 0.05, ** *p* ≤ 0.01.

To confirm the infectivity of GFP-labelled *T. gondii* tachyzoites, PBMCs were either untreated, treated with live tachyzoites, or treated with heat-killed (H/K) tachyzoites. FACS analysis of GFP+ cells revealed that approximately 45% of live PBMCs were infected 24 h following treatment with live tachyzoites. No cells in the untreated or heat-killed tachyzoite groups were positive for GFP signals (Figure 4A,B). Approximately 93% of infected, GFP+ PBMCs were viable according to fixable viability dye staining (data not shown). The successful infection of a large proportion of cells without excessive cell death indicated that both the infective dose of 1:1 MOI, and the incubation time of 24 h were appropriate.

#### 3.3.2. In Response to *T. gondii* Infection, Monocytes Produce Low Levels of IL-12p40

Porcine monocytes and moDC were stimulated with live or heat-killed *T. gondii* tachyzoites, or with the recombinant *T. gondii* antigen profilin, and IL-12p40 and TNF-α concentrations in cell culture supernatants were assessed. We observed a complete lack of detectable TNF-α response from both monocytes and moDC following infection with live tachyzoites, as well an absent IL-12 response from moDC. Porcine monocytes did appear to slightly upregulate IL-12 following live tachyzoite stimulation; however, responses were heterogenous, as monocytes from two of five pigs did not produce detectable levels of IL-12p40 (Figure 4C). No IL-12p40 or TNF-α production was observed following stimulation with heat-killed tachyzoites or recombinant profilin (Figure 4C).

#### 3.3.3. Neither cDC nor pDC Produce IL-12 following *T. gondii* Infection

IL-12p40 and TNF-α production, and costimulatory molecule expression represented by CD80/86, were analyzed in cDC subsets and pDC following stimulation with live tachyzoites. Cell viability was assessed, as well as the percentage of GFP+ *T. gondii*-infected cells. In contrast to our observations following TLR ligation, no upregulation of IL-12p40 or TNF-α was observed from cDC or pDC following *T. gondii* exposure, and CD80/86 upregulation was much less pronounced (Figure 4D). However, the percentages of infected DC were far lower than observed in whole PBMCs and varied substantially between subsets (Figure 4E,F). These data indicated that extensive parasite clearance by DC may be occurring, with cDC1 being particularly efficient.

#### 3.3.4. CD3– NKp46+ Cells Do Not Produce IL-12 following *T. gondii* Infection

**Figure 5:**
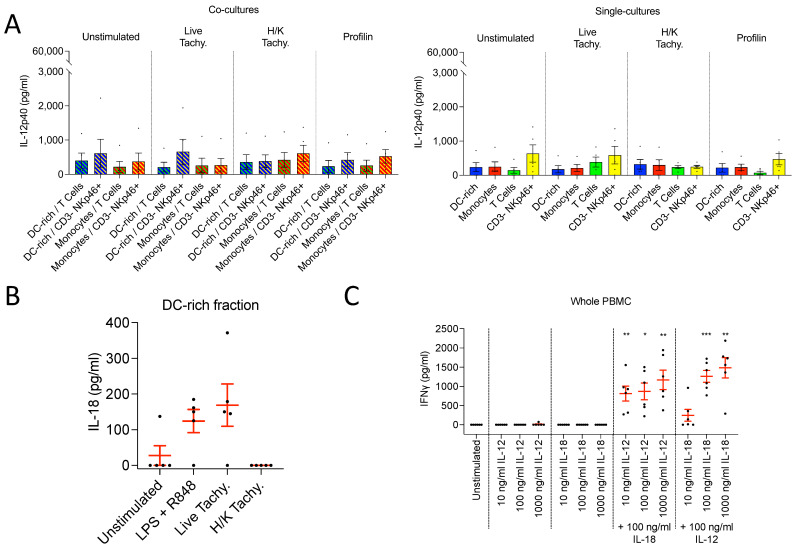
(**A**) IL-12p40 concentrations in the supernatants of porcine monocytes and DC-enriched PBMCs, in isolation and co-culture with T cells or CD3– NKp46+ cells, following 24 h stimulation with live and heat-killed *T. gondii* tachyzoites and recombinant profilin. Stimulations were performed with *n* = 5 different pigs. All cell types were stimulated in parallel from each pig. (**B**) Concentrations of IL-18 in the cell culture supernatants of DC-enriched PBMCs, following 24 h stimulation with TLR ligands or live or heat-killed T. gondii tachyzoites. Stimulations were performed with *n* = 5 different pigs. (**C**) IFNγ concentrations in the supernatants of cultured PBMCs following 48 h stimulation with different concentrations of recombinant IL-12p70 and/or IL-18, *n* = 6. Asterisks indicate statistical significance; paired *t* test; * *p* ≤ 0.05, ** *p* ≤ 0.01, *** *p* ≤ 0.001.

Monocytes and DC-enriched PBMC were co-cultured with CD3+ T cells and CD3– NKp46+ cells, and stimulated with live and heat-killed tachyzoites, and recombinant profilin. The lack of IL-12 response observed by single-cultured porcine myeloid cells following *T. gondii* stimulation was not altered by co-culture with T cells. Interestingly we found that the CD3– NKp46+ cells, despite producing a strong IL-12 response to TLR ligation as demonstrated, did not respond to *T. gondii* exposure (Figure 5A). Further ELISA data demonstrated that CD3– NKp46+ cells did not upregulate IFNγ production either following *T. gondii* stimulation, suggesting perhaps an inability to recognize the parasite (data not shown).

#### 3.3.5. DC-enriched PBMC Produce IL-18 following *T. gondii* Infection

While IL-12 is vital for the differentiation of naïve CD4+ T cells into IFNγ-producing Th1 cells, it has been demonstrated in mice that IL-18 can induce IFNγ production from Th1 cells. Further, IL-18 and IL-12 act synergistically to induce IFNγ production by murine NK cells, B cells, and myeloid cells [29]. Here, we observed a lack of detectable IL-12 production by porcine DC, and a weak and heterogenous IL-12 response from porcine monocytes, following *T. gondii* stimulation. Due to this, and the previously described role of IL-18 in IFNγ induction in mice, we next examined whether IL-18 is involved in inducing IFNγ production in pigs following *T. gondii* exposure.

Interestingly, we observed a notable upregulation of IL-18 by DC-enriched PBMC, 24 h after TLR ligation with LPS + R848 or stimulation with live, but not heat-killed, *T. gondii* tachyzoites (Figure 5B). IL-12p40 production was observed only in the TLR-stimulated cultures (data not shown). This data suggested that IL-18 may indeed play a role in immune response initiation following *T. gondii* infection in pigs.

### 3.4. IFNγ Response of Porcine PBMC following IL-12 and IL-18 Stimulation

#### 3.4.1. IL-12 and IL-18 Stimulation in Combination, but Not Alone, Induces IFNγ Production

To verify the IFNγ-inducing capacity of IL-18 on porcine cells, we analyzed the efficacy of recombinant IL-12p70 and IL-18 to induce IFNγ production. Whole PBMCs were stimulated with different concentrations of IL-12p70 and IL-18, alone and in combination, to assess the relative contribution of each cytokine for IFNγ induction. When administered alone, neither cytokine induced IFNγ production. However, when combined, substantial quantities of IFNγ were detected. Increasing the concentration of either cytokine resulted in a corresponding increase in the quantity of IFNγ produced, confirming a dose-dependent response. However, IFNγ production varied more greatly when IL-18 concentrations were altered than when IL-12p70 concentrations were varied. Thus, while the presence of both cytokines is essential for IFNγ production in the pig, IL-18 seems to play a greater role in determining the magnitude of the IFNγ response (Figure 5C).

#### 3.4.2. IFNγ Produced following IL-12 and IL-18 Stimulation Is Derived from NK Cells

**Figure 6:**
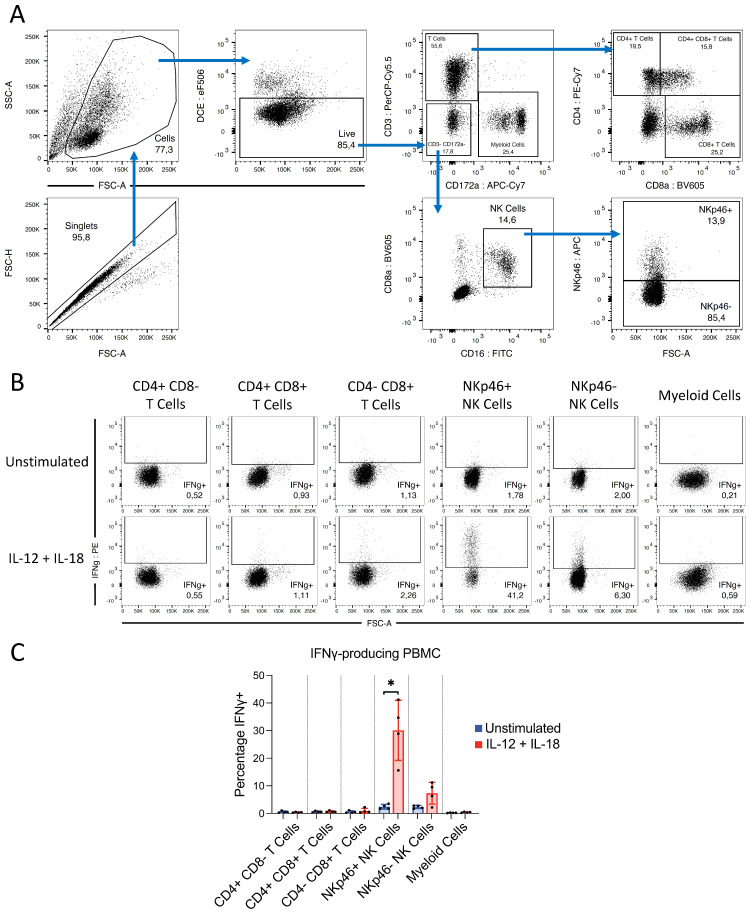
(**A**) Gating strategy used for PBMC subsets prior to analysis for IFNγ production. Cell aggregates and debris were excluded, followed by gating on DCE− live cells. The CD3+ CD172a− population consisted of T cells, which were further CD8−, CD4+ CD8+, and CD4− CD8+ subpopulations. The CD3– CD172a− population contained NK cells, which were identified by coexpression of CD8a and CD16, and further divided into NKp46+ and NKp46− subpopulations. The CD3– CD172a+ population consisted of myeloid cells. (**B**) Representative staining illustrating upregulation of IFNγ expression by NKp46+ and NKp46− NK cells following 12 h stimulation with recombinant IL-12p70 and IL-18. (**A**,**B**) FACS plots displaying forward and side-scatter values on a linear scale, while all fluorescent markers are displayed on a biexponential scale. (**C**) Percentage IFNγ+ cells within T-cell and NK-cell subsets and myeloid cells. The analysis was performed with *n* = 4 pigs. Asterisks indicate statistical significance; paired *t* test; * *p* ≤ 0.05.

We next aimed to identify the PBMC subsets responsible for IFNγ production in the pig following IL-12 and IL-18 stimulation. Therefore, PBMCs were stimulated with IL-12p70 and IL-18, and IFNγ production was examined by FACS. IFNγ production was greatly upregulated by NK cell subsets following treatment, while T cells and myeloid cells did not respond. Within the NK cell population, the NKp46+ subpopulation exhibited the greatest production of IFNγ, with approximately 30% of cells being IFNγ+ following IL-12p70/IL-18 stimulation. The NKp46− NK cell subset responded to a lesser extent, with approximately 7% of cells producing IFNγ. These data indicated that NK cells, and especially NKp46+ NK cells, are responsible for IFNγ production by porcine PBMC following IL-12 and IL-18 stimulation (Figure 6).

## 4. Discussion

In the current study, we demonstrated that porcine pDC are the only bona fide DC subset in PBMCs that produce IL-12p40 or TNF-α following different TLR ligations in vitro. Porcine monocytes and moDC did not produce IL-12 following TLR stimuli, although they did produce TNF-α. These findings corresponded with data published by Auray et al. (2016) [25] that demonstrated a lack of upregulation of TNF-α or IL-12p40 secretion by porcine cDC1 and cDC2 following stimulation with multiple TLR ligands. Their panel included LPS, poly(I:C), and gardiquimod, a synthetic TLR 7 ligand derived from imidazoquinoline, structurally highly similar to the TLR 7/8 ligand R848 used by us, another imidazoquinoline derivative. In line with our data, they observed significant production of these cytokines only by pDC.

In contrast, neither IL-12p40 nor TNF-α was produced by any porcine DC following live *T. gondii* tachyzoite exposure. Likewise, monocytes did not express TNF-α following live tachyzoite stimulation, although we detected a weak and heterogenous IL-12 response from monocytes. Thus, a major difference between the responses to TLR ligation and protozoan parasite infection exists in pigs. Interestingly, a recent study published by Cui et al. (2020) [30] found no upregulation of IL-12p35 or p40 mRNA by porcine alveolar macrophages following stimulation with *T. gondii* tachyzoites. In combination with our findings in DC and monocytes, this data suggests that IL-12 is not produced in large quantities by porcine myeloid cells following *T. gondii* exposure. The authors did, however, uncover upregulation of TNF-α mRNA in macrophages following tachyzoite exposure, indicating the comparative lack of IL-12 protein production observed was not due to an inability to detect the parasite infection.

We found that the percentage of infected DC was low following live tachyzoite stimulation in comparison to PBMCs that received the same treatment. Both experiments consisted of multiple repetitions performed on different days, each with a fresh batch of tachyzoites. It is therefore improbable that tachyzoite infectivity simply varied between experiments. Channon et al. (2000) [31] examined the infectivity of PLK strain *T. gondii* tachyzoites in human PBMC subsets, and found that infectivity varied considerable between cell types. Following 24 h incubation at 1:1 MOI, approximately 50% of human monocytes and 25% of moDC contained at least 1 tachyzoite, compared with 5% of lymphocytes. The relative infectivity of *T. gondii* in bona fide DC has not been previously examined in pigs or other species; however, our data in pigs indicated it is low compared with whole PBMCs. Although resistance to invasion may contribute to this phenomenon, the low percentages of infected DC may also be the result of extensive parasite clearance, with parasite-clearance capacity varying by DC subset. We did not observe large differences in the percentages of live cells between live tachyzoite treated and negative control groups, implying that the difference was not simply due to extensive cell death of infected DC.

IL-12 production by porcine monocytes, moDC, and cDC was not observed following TLR ligation. In contrast, we observed a very strong upregulation of IL-12p40 expression by porcine CD3– NKp46+ cells following stimulation with LPS or poly(I:C) and R848. Walzer et al. (2007) [32] examined expression of NKp46 in humans, seven strains of mouse, and three monkey species. They demonstrated that NKp46 is expressed almost exclusively by NK cells in all of these species, although a small subpopulation of CD3+ NKp46+ γδ T cells was observed in mice. Virtually all NK cells tested positive for NKp46 in all cases. On the weight of this evidence, as well as earlier studies describing NKp46 as an NK cell-specific marker in rats [33] and cattle [34], they proposed ‘CD3– NKp46+’ as a pan-mammalian NK cell-specific phenotype. This paradigm was further supported by subsequent findings in sheep [35]. Mair et al. (2012) [36] examined in detail the expression of NKp46 in pigs, and found that porcine CD3– NKp46+ lymphocytes exhibit a classical NK cell phenotype. However, they also found another population of NKp46− NK cells that displayed comparable cytolytic activity, but reduced IFNγ production capacity.

To our knowledge, our finding that porcine CD3– NKp46+ cells express IL-12p40 following stimulation has not previously been documented. Given the previous characterization of NKp46+ cells in pigs, we appear to have identified endogenous IL-12 production by porcine NK cells. We cannot, however, exclude the possible existence of porcine CD3– NKp46+ myeloid cells, since most phenotyping of CD3– NKp46+ cells by Mair et al. (2012) [36] included pregating on lymphocytes according to size and granularity. Despite this, they did demonstrate a lack of NKp46 and CD14 coexpression, gating on lymphoid and myeloid cells, thus ruling out all previously described porcine monocyte subsets [37]. The phenotype of IL-12-producing CD3– NKp46+ cells will be further investigated in future experiments.

Data published by De Pelsmaeker et al. (2017) [38] suggested that porcine NK cells display numerous features associated with antigen-presenting cells. This includes expression of MHCII and costimulatory molecules, activation of T cells, and either phagocytosis or macropinocytosis. MHCII and costimulatory molecule expression, and T-cell activation capacity, were both shown to be upregulated by porcine NK cells following stimulation with IL-12 and IL-18, and especially with the addition of IL-2. However, endogenous cytokine production capacity following antigen stimulation was not examined. IL-12 production by porcine NK cells would further underline the importance of NK cells as APC-like cells in pigs, and may compensate for low IL-12 production by myeloid cells.

Although we identified strong upregulation of IL-12 production by CD3– NKp46+ cells following TLR ligation, as with pDC, IL-12p40 was not produced by CD3– NKp46+ cells following stimulation with *T. gondii* tachyzoites. Experiments in mice by Hou et al. (2011) [39] have previously illustrated that until day 5 post-*T. gondii* infection, IFNγ-producing cells are primarily NK cells. Notably, IFNγ production by NK cells was almost completely absent when MyD88 was selectively knocked out of DC, impairing their ability to produce IL-12, but was revived by administration of recombinant IL-12. A strong upregulation of IL-12, as seen in other species, was not detected in any of our isolated or co-cultured porcine cell populations following stimulation with *T. gondii*. However, we identify an upregulation of IL-18 production by DC-enriched PBMC following stimulation with TLR ligands or live *T. gondii* tachyzoites, but not heat-killed tachyzoites. This is reminiscent of the IL-12 response of human DC, which likewise respond to TLR ligation and live tachyzoite exposure, but not stimulation with heat-killed tachyzoites [6].

Since IL-12 and IL-18 have been shown to synergistically induce IFNγ production in the mouse [29], we investigated the relative contribution of these two cytokines for IFNγ induction in the pig. We found that IFNγ was not produced in detectable quantities by porcine PBMC following stimulation with either cytokine individually, even when administered at a high concentration. However, when combined, IFNγ production was greatly upregulated—with this IFNγ originating from NK cells. Corroborating previous findings by Mair et al. (2012) [36], NKp46+ NK cells exhibited a more potent IFNγ response than NKp46− NK cells, although IFNγ was still upregulated to a lesser degree within the NKp46− NK cell population.

We demonstrated that IL-18 alone was not capable of inducing IFNγ production. However, we observed that the concentration of IL-18 applied had a greater impact on controlling the magnitude of the IFNγ response than the concentration of IL-12. This corresponds to findings in mice showing that IL-12 and IL-18 synergistically induce TCR-independent IFNγ production by T cells, with IL-18 most strongly controlling the magnitude of the IFNγ response [40]. Therefore, the production of IL-18 following live *T. gondii* tachyzoite exposure is likely important for upregulating the innate IFNγ response.

We did observe low levels of IL-12p40 production by porcine monocytes following live tachyzoite exposure. Therefore, this may be sufficient for inducing Th1 polarization, and IFNγ production by NK cells in conjunction with IL-18. However, it could also be the case that the migration of tachyzoites through the intestinal epithelium following oral infection disturbs its barrier function and allows intestinal bacteria reach the lamina propria. This would likely be sufficient to induce much higher levels of IL-12, similar to the responses observed here by pDC and CD3– NKp46+ cells following TLR ligation.

Whether transmigration of *T. gondii* through the intestinal epithelial layer results in disturbance of barrier function remains controversial [41]. Recently published data by Holthaus et al. (2021) [42] indicates that *T. gondii* infection does not disrupt the epithelial barrier function of murine intestinal organoid-derived monolayers. However, Benson et al. (2009) [43] demonstrated that pretreatment with antibiotics to deplete the gut microbiota prior to oral *T. gondii* infection considerably reduced the resulting IL-12 response in mice. Further, TLR 11 knockout mice did not produce IL-12 following intraperitoneal *T. gondii* infection, but did produce IL-12 following oral infection, with this IL-12 production being abolished following antibiotic pretreatment. Therefore, mice exhibit an additional microbiota-dependent response to oral *T. gondii* infection that is independent of TLR 11-mediated recognition. Similar pathways may be particularly important in pigs and humans, in which TLR 11 is nonfunctional. Species-specific differences in intestinal barrier disruption may also be present, with Briceno et al. (2016) [44] describing significant loss of barrier integrity in the Caco-2 human colorectal adenocarcinoma cell line. Whether porcine intestinal barrier function is disturbed during *T. gondii* transmigration is not yet known. However, the intestinal microbiota may play an important role in immune response initiation during oral *T. gondii* infection, and compensate for the comparatively weak IL-12 responses observed in the pig following tachyzoite stimulation alone.

To further elucidate the mechanisms responsible for innate IFNγ induction in the pig following oral *T. gondii* infection, in vivo studies would be beneficial. This would allow investigation of the importance of complex factors such as the host microbiota in enhancing induction of IL-12 production following infection. In vivo studies would enable not only further elucidation of the mechanisms of immune initiation in the pig following *T. gondii* infection, but also analysis of the resulting adaptive T-cell response. Porcine *T. gondii*-specific T cells could be examined [45] in blood and tissues during acute and chronic infection. This would shed new light on the porcine *T. gondii*-specific T-cell response, and enable assessment of the relevance of the pig as a human-like model of adaptive immunity to *T. gondii* infection.

Postnatal *T. gondii* infections in pigs usually remain asymptomatic, and a strong antigen-specific IFNγ response as observed in other species has also been demonstrated in pigs. Therefore, it is clear that pigs are able to respond effectively to *T. gondii* infection. However, here we demonstrated that early immune initiation following in vitro *T. gondii* tachyzoite exposure was considerably different than that reported in mice and humans. Murine cDC1 and inflammatory monocytes, and human cDC2 and intermediate and nonclassical monocytes, produce a potent IL-12 response immediately following *T. gondii* tachyzoite stimulation. In pigs, DC do not produce IL-12 in response to tachyzoite exposure, and monocytes produce a very weak and heterogenous response. However, IL-18 is produced by porcine DC-enriched PBMC following live tachyzoite infection, which, as we demonstrated, plays an important role in IFNγ induction by NK cells. Additionally, we identified CD3– NKp46+ cells as an important source of IL-12p40 in the pig following TLR ligation, which to our knowledge has not been previously reported in pigs or other species.

## Supplementary Materials

The following are available online at https://www.mdpi.com/article/10.3390/microorganisms9091828/s1, Table S1: Additional details of all antibodies used.
