# Supplementary material for: Early Immune Initiation by Porcine Cells following Toxoplasma gondii Infection versus TLR Ligation
Source: Microorganisms. 2021 Aug 28;9(9):1828. doi: 10.3390/microorganisms9091828 (PMC8471494; doi:10.3390/microorganisms9091828)
Supplement: Supplementary file 1 [file microorganisms-09-01828-s001.zip › microorganisms-1303793-supplementary.pdf]

Target	Clone	Reactive Species	Host Species	Isotype	Conjugate	Manufacturer	Product Code
CD3e	PPT3	Pig	Mouse	IgG1	-	Bio-Rad	MCA5951GA
gdTCR1	PGBL22A	Pig	Mouse	IgG1	-	KingFisher	WS0621S-100
CD335/NKp46	VIV-KM1	Pig	Mouse	IgG1	-	Bio-Rad	MCA5972GA
CD21	BB6-11C9.6	Pig	Mouse	IgG1	-	Biozol	SBA-4530-01
IgM	F008-1629	Pig	Mouse	IgG1	-	BD Biosciences	552551
IgG1	X-56	Mouse	Rat	IgG1	MACS MicroBeads	Miltenyi Biotech	130-047-101
CD14	TÜK4	Human (Cross-reactive to pig [19])	Mouse	IgG2a	MACS MicroBeads	Miltenyi Biotech	130-050-201
IgG1	RMG1-1	Mouse	Rat	IgG	APC-Cy7	Biolegend	406620
CD14	MIL2	Pig	Mouse	IgG2b	-	Bio-Rad	MCA1218GA
IgG2b	Polyclonal	Mouse	Goat	-	APC-Cy7	Southern Biotech	SBA-1090-19
CADM1	3E1	Human (Cross-reactive to pig [19])	Chicken	IgY	Alexa 647	Biozol	MBL-CM004-A64
CD4a	74-12-4	Pig	Mouse	IgG2b	PerCP-Cy5.5	BD Biosciences	561474
CD172a	74-22-15A	Pig	Mouse	IgG2b	PE	BD Biosciences	561498
CD80/86	CD152 (fusion protein)	Human (Cross-reactive to pig [19])	Mouse (Fc region)	IgG2a	-	Ancell	ANC-501-820
IgG2a	R19-15	Mouse	Rat	IgG1	Brilliant Violet 605	BD Biosciences	564024
IL-12/IL-23p40	116211	Pig	Mouse	IgG2b	Biotin	R&D Systems	BAM9122
TNF-a	MAb11	Human (Cross-reactive to pig [19])	Mouse	IgG1	Pacific Blue	Biolegend	502920
Streptavidin	-	-	-	-	PE-Cy7	ThermoFisher	25-4317-82
CD172a	BL1H7	Pig	Mouse	IgG1	-	Bio-Rad	MCA2312GA
CD8a	76-2-11	Pig	Mouse	IgG2a	-	ThermoFisher	MA528717
CD3	BB23-8E6-8C8	Pig	Mouse	IgG2a	PerCP-Cy5.5	BD Biosciences	561478
CD4	74-12-4	Pig	Mouse	IgG2b	PE-Cy7	BD Biosciences	561473
CD16	G7	Pig	Mouse	IgG1	FITC	Bio-Rad	MCA1971F
CD335/NKp46	VIV-KM1	Pig	Mouse	IgG1	APC	Bio-Rad	MCA5972APC
IFNg	P2G10	Pig	Mouse	IgG1	PE	BD Biosciences	559812
